# Cost-effectiveness of a Multicomponent Intervention for Hypertension Control in Low-Income Settings in Argentina

**DOI:** 10.1001/jamanetworkopen.2021.22559

**Published:** 2021-09-14

**Authors:** Yichen Zhang, Lei Yin, Katherine Mills, Jing Chen, Jiang He, Alfredo Palacios, Andrés Pichon Riviere, Vilma Irazola, Federico Augustovski, Lizheng Shi

**Affiliations:** 1 Department of Health Policy and Management, School of Public Health and Tropical Medicine, Tulane University, New Orleans, Louisiana; 2 Department of Epidemiology, School of Public Health and Tropical Medicine, Tulane University, New Orleans, Louisiana; 3 Institute for Clinical Effectiveness and Health Policy, Buenos Aires, Argentina; 4 School of Public Health, University of Buenos Aires School of Medicine, Buenos Aires, Argentina; 5 CONICET (National Scientific and Technical Research Council), Buenos Aires, Argentina

## Abstract

**Importance:**

Hypertension is highly prevalent in low- and middle-income countries, and it is an important preventable risk factor for cardiovascular diseases (CVDs). Understanding the economic benefits of a hypertension control program is valuable to decision-makers.

**Objective:**

To evaluate the long-term cost-effectiveness of a multicomponent hypertension management program compared with usual care among patients with hypertension receiving care in public clinics in Argentina from a health care system perspective.

**Design, Setting, and Participants:**

This economic evaluation used a Markov model to estimate the cost-effectiveness of a hypertension management program among adult patients with uncontrolled hypertension in a low-income setting. Patient-level data (743 individuals for multicomponent intervention; 689 for usual care) from the Hypertension Control Program in Argentina trial (HCPIA) were used to estimate treatment effects and the risk of CVD. Three health states were included in each strategy: (1) low risk of CVD, (2) high risk of CVD, and (3) death. The total time horizon was the lifetime, and each cycle lasted 6 months.

**Main Outcomes and Measures:**

Model inputs were based on trial data and other published sources. Cost and utilities were discounted at a rate of 5% annually. The incremental cost-effectiveness ratio (ICER) between the multicomponent intervention and usual care was calculated using the difference in costs in 2017 international dollars (INT $) divided by the difference in effectiveness in quality-adjusted life-years (QALYs). One-way sensitivity analysis and probabilistic sensitivity analysis were performed to assess the uncertainty and robustness of the results.

**Results:**

In the original trial, the 743 participants in the intervention group (349 [47.0%] men) had a mean (SD) age of 56.2 (12.0) years, and the 689 participants in the control group (311 [45.1%] men) had a mean (SD) age of 56.2 (11.7) years. In the base-case analysis, the HCPIA program yielded 8.42 discounted QALYs and accrued INT $3096 discounted costs, while usual care yielded 8.29 discounted QALYs and accrued INT $2473 discounted costs. The ICER for the HCPIA program was INT $4907/QALY gained. The model results remained robust in sensitivity analyses, and the model was most sensitive to parameters of program costs.

**Conclusions and Relevance:**

In this study, the HCPIA multicomponent intervention vs usual care was a cost-effective strategy to improve hypertension management and reduce the risk of associated CVD among patients with hypertension who received services at public clinics in Argentina. This intervention program is likely transferable to other settings in Argentina or other lower- and middle-income countries.

## Key Points

**Question:**

Is a multicomponent hypertension control intervention in low-income settings in Argentina cost-effective in the long term?

**Findings:**

In this economic evaluation, compared with usual care, a multicomponent hypertension control intervention was cost-effective in a lifetime horizon in Argentina from a health care system perspective.

**Meaning:**

These findings suggest that a multicomponent hypertension control intervention may be transferrable to other settings in Argentina or other low- and middle-income countries.

## Teaser

This economic evaluation examines the long-term cost-effectiveness of a multicomponent hypertension management program compared with usual care among patients with hypertension receiving care in public clinics in Argentina from a health care system perspective.

## Introduction

Hypertension is a major global public health challenge because of its high prevalence.^1,2^ With low awareness or lack of control of hypertension, the prevalence of hypertension is growing particularly quickly in low- and middle-income countries (LMICs), such as those in Latin America and the Caribbean.^3,4^ In 2017, hypertension prevalence among adults in Argentina was 36.3%, awareness of it was 61%, and blood pressure (BP) control (ie, systolic BP [SBP] <140 mm Hg; diastolic BP [DBP], <90 mm Hg) was 24%.^5^ Hypertension is the most important preventable risk factor for cardiovascular diseases (CVDs) including stroke, coronary artery disease, heart failure, atrial fibrillation, and peripheral vascular disease, which are the leading causes of death.^6,7^ In a meta-analysis of more than 1 million adults, the risk of having a CVD began to rise in all age groups with SBP greater than 115 mm Hg and DBP greater than 75 mm Hg.^8,9^ For every 20 mm Hg higher SBP and 10 mm Hg higher DBP, the risk of death from heart disease or stroke doubles.^10^ Inadequate public health financing and limited availability of health care facilities in LMICs might necessitate more attention to hypertension control. In Argentina, 6.1% of deaths from diseases of the circulatory system were attributed to hypertensive disease, with a specific mortality rate of 13.8 per 100 000 individuals.^11,12^ Therefore, the prevention and management of high BP is currently a major public health challenge, and it is especially important to develop effective, affordable, and sustainable programs for hypertension control in Argentina.

The Hypertension Control Program in Argentina (HCPIA) was an 18-month cluster randomized trial (NCT01834131) to test whether a multicomponent intervention program conducted primarily within a national public primary care system would improve hypertension control among patients with low income and hypertension in Argentina.^13,14^ The multicomponent intervention included a community health worker (CHW)–led home intervention (health coaching, home BP monitoring, and BP audit and feedback), a physician intervention, and a text-messaging intervention over 18 months.^13^ Compared with other intervention programs for hypertension control, this trial focused on adopting the CHW-led intervention, which is a more affordable and sustainable approach for low-income settings. The trial results showed that at the end of the intervention, this multicomponent intervention, compared with usual care, significantly reduced SBP (6.6 mm Hg [95% CI, 4.6-8.6 mm Hg]; *P* < .001) and DBP (5.4 mm Hg [95% CI, 4.0-6.8 mm Hg]; *P* < .001) among patients with hypertension receiving care in public clinics in Argentina.^13^

Despite the clinical benefits, there is an increasing worldwide concern about the economic burden of hypertension and associated cardiovascular outcomes. Thus, understanding the clinical and economic benefits of hypertension control programs in LMICs will provide valuable evidence to clinicians, health care professionals, and other health care decision-makers. The previous trial-based short-term cost-effectiveness analysis showed that the 18-month CHW-led comprehensive approach used in the HCPIA trial was cost-effective compared with usual care for BP control.^15^ Quality-adjusted life-years (QALYs) significantly increased by 0.06 (95% CI, 0.04-0.09) in the intervention group, and the SBP net difference favored the intervention group by 5.3 mm Hg (95% CI, 0.27-10.34 mm Hg).^16^ The incremental cost-effectiveness ratio (ICER) was US $3299 per QALY and US $26 per mm Hg of SBP. Although the economic benefit was quantified through the short-term cost-effectiveness analysis, it is unknown whether these findings are likely to translate to a longer time horizon, as recommended in many economic evaluation guidelines.^17,18,19,20^ The aim of this study was to assess the long-term cost-effectiveness of this multicomponent hypertension management program compared with usual care among patients with low income and hypertension in Argentina from a health care system perspective.

## Methods

The institutional review boards of Tulane University and Hospital Italiano de Buenos Aires approved the study. Informed consent was signed by all participants during screening. The economic modeling approach included in this study followed the International Society for Pharmacoeconomics and Outcomes Research (ISPOR) Good Research Practice guideline.^21,22,23^

### Trial Population and Study Design

The HCPIA trial was conducted among 18 primary health care centers within a national public system in Argentina, and the eligible population included adult patients (aged ≥21 years) with hypertension and uncontrolled BP (SBP ≥140 mm Hg and/or DBP ≥90 mm Hg on ≥2 separate baseline visits) and their adult family members living in the same household. Cluster randomization was stratified by geographic region, and primary health care centers were randomly assigned to the control or intervention group. A total of 743 patients from 9 centers were randomized to receive the multicomponent intervention, and a total of 689 patients from the other 9 centers were randomized to usual care (control group) without any active study intervention.

The 18-month multicomponent intervention included health care practitioner education, a CHW-led home-based intervention, and a text-messaging health intervention among patients and their families. Health care practitioner education included a 2-day interactive training session followed by onsite field testing and certification. The family-based intervention started with an initial 90-minute home visit and was followed by subsequent 60-minute monthly or bimonthly follow-up visits, during which CHWs would provide tailored counseling to participants and their families on lifestyle modification, home BP monitoring, and medication adherence skills. All patients in the intervention group were given an automatic home BP monitor and log and were trained to record their BP weekly. Individualized text messages were sent to participants weekly to promote lifestyle changes and remind them about medication adherence.

Standard questionnaires and measurement methods were used to collect study data at baseline and 6, 12, and 18 months of follow-up. The primary outcome was difference in net change in SBP and DBP from baseline to month 18 between the multicomponent intervention and control groups. Other trial details as well as details of the trial-based economic evaluation have been published previously.^13,14,15^

### Model Structure

**Figure 1.**
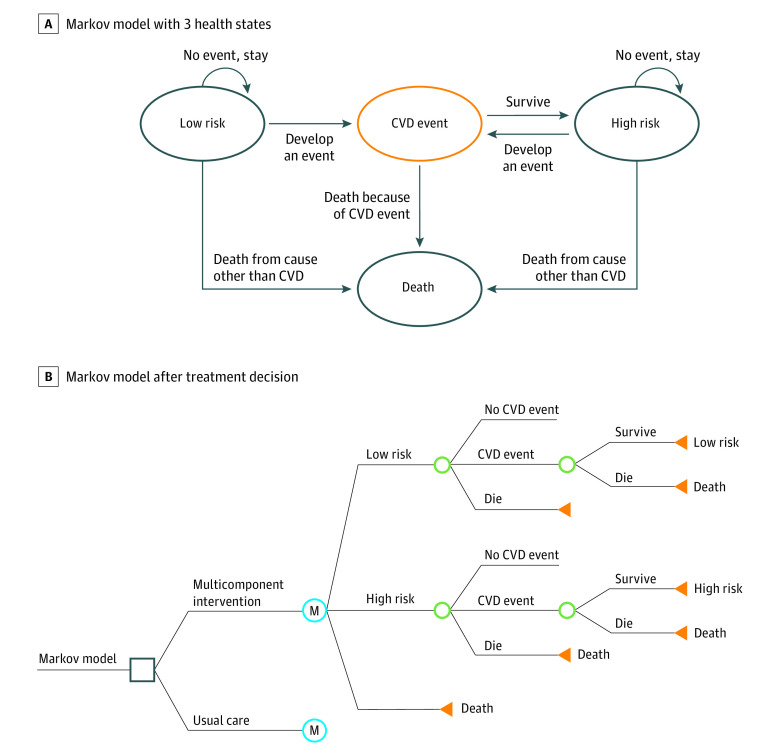
Diagram of the Markov Model B, The square indicates the decision node, the point at which a treatment strategy is chosen; the letter M indicates the Markov node, with branches indicating the health states in transition within every 6 months; the green circles indicate the chance node, after which there is a probability of the occurrence of each event; and the orange triangle indicates the terminal node, the end of a pathway within a 6-month cycle.

The Markov model was structured around disease states and incorporated aggregated patient-level data derived from the HCPIA trial (Figure 1). The model compared the 2 strategies included in the trial: a multicomponent hypertension management program (ie, intervention group) vs usual care (ie, control group). The model included 3 health states: (1) low risk of CVD, (2) high risk of CVD, and (3) death. A composite CVD outcome was defined as CVD events including myocardial infarction and stroke, among others. Independent of the treatment option, individuals can move between states or stay in the low-risk or high-risk states, with death being the absorbing health state. All study participants entered the corresponding health states in the model at the same time (ie, the beginning of the trial) with their mean age assumed to be 55 years. Due to the chronic condition of hypertension, a lifetime horizon of 45 years was applied, assuming a total life expectancy of 100 years. Thus, the model included an 18-month trial period, with the remaining 43.5 years as the posttrial period. Each cycle length was 6 months. At the end of each cycle, the cohort was redistributed to 1 of 3 health states depending on the events of the previous cycle. Regardless of baseline risk, patients who survived a CVD event started a new cycle in the high-risk group, according to a similar Markov structure adopted in the previous study.^24^ The main model outcomes included QALYs; total costs, including multiple components (ie, intervention program cost, CVD event costs, and follow-up costs); and incremental cost-effectiveness ratios (ICERs) comparing the HCPIA intervention with usual care. Model parameters were varied in the sensitivity analyses.

### Costs

The cost of the intervention program was calculated based on the trial data. A linear change was assumed over 18 months to estimate the cost per patient at 6 months (Table 1). In detail, the study included 2 main cost categories from the health care system perspective: the costs of implementing the intervention itself and the costs associated with the utilization of health services by individuals in both intervention and control groups.^15^ The fixed and varied costs of implementing the intervention were included. The fixed costs included the development and maintenance of an online platform that contributed to the management of the intervention and generated and sent customized text messages promoting a healthy lifestyle to participants. The varied costs included costs for training activities for CHWs on the participant intervention; training activities for physicians focused on standard treatment algorithms for stepped-care management based on clinical guidelines; BP monitors for all patients with hypertension for weekly home measurement; the number of hours spent by CHWs on education, motivation, social support, and promoting health care utilization for participants and their families; the number of hours spent by the CHW coordinator; and the number of text messages sent to each participant.

**Table 1.** Intervention and Nonintervention Costs for 6 Months

Services	Cost per patient, 2017 INT $	
	Intervention group	Control group
Intervention		
Platform development and maintenance	3.43	NA
Training workshop	2.01	NA
Patient educational material	3.05	NA
Self-monitoring blood pressure	9.12	NA
Community health workers visit	30.56	NA
Field work coordination	1.94	NA
Text messages	3.77	NA
Subtotal	53.88	NA
Health services not related to intervention within 6 mo		
Hospitalization within 6 mo	8.18	5.64
Hospitalization LOS in general ward	20.48	14.27
Hospitalization in CU or ICU	2.84	13.07
Outpatient care and testing	14.87	8.52
Antihypertensive medications	58.93	41.18
Subtotal	105.30	82.69
Total	159.18	82.69

Abbreviations: CU, coronary unit; ICU, intensive care unit; INT $, international dollars; LOS, length of stay; NA, not applicable.

The costs of health care service utilization were calculated using the utilization rate of each health care resource at the patient level and its associated unit cost in each province. The use of both outpatient and inpatient services (eg, visits, medications, laboratory studies, hospitalizations) was recorded through specific questionnaires administered at baseline and 6, 12, and 18 months. If follow-up questionnaire data on the utilization of a specific health service were missing, it was assumed that the utilization rate of this service was equal to that reported in the previous questionnaire. Other details in terms of intervention cost calculation and derivation were presented in our previous short-term within-trial cost-effectiveness publication.^15^ Other cost inputs included costs of each CVD event. Follow-up costs for CVD events were obtained from the literature and inflated to 2017 international dollars (INT $) (Table 2).^8,15,24,25,26,28,29^ Costs were reported in international dollars using the purchasing power parities (PPP) conversion rate suggested by the International Monetary Fund. The PPP conversion rate was 11.47 Argentinian pesos per international dollar in 2017.^30^

**Table 2.** Summary of Input Parameters for the Model

Name	Description	Base case (range)^a^	Distribution type	Source
Cost, INT $				
CVD event	Costs of each CVD event	1732.47 (1299.35 to 2165.58)	Gamma	Perman et al,^24^ 2011
CVD event follow-up	Follow-up costs for CVD event	300.00 (225.00 to 375.00)	Gamma	Gaziano et al,^25^ 2014
Health service, control	Cost of health service for control group	82.69 (62.02 to 103.36)	Gamma	HCPIA trial^13,15^
Intervention	Cost of intervention program	53.88 (40.41 to 67.35)	Gamma	HCPIA trial^13,15^
Health service, intervention	Cost of health service for intervention group	105.30 (78.98 to 131.63)	Gamma	HCPIA trial^13,15^
Transition probability				
High risk to CVD event	Probability patient in intervention group with high risk has CVD event	0.0267 (0.0201 to 0.0334)	Beta	Framingham equation,^26^ based on HCPIA trial data
Low risk to CVD event	Probability patient in intervention group with low has CVD event	0.0058 (0.0044 to 0.0073)	Beta	Framingham Equation,^26^ based on HCPIA trial data
CVD event to death	Probability of CVD event being fatal	0.30 (0.26 to 0.33)	Triangular	Rosendaal et al,^27^ 2016
Utility				
High risk	Quality-of-life weight for high-risk disease state	0.7963 (0.5972 to 0.9954)	Beta	HCPIA trial^13,15^
Low risk	Quality-of-life weight for low-risk disease state	0.8176 (0.6132 to 1.0000)	Beta	HCPIA trial^13,15^
CVD	Quality-of-life weight in the CVD event state, within 1 year	–0.2775 (–0.2081 to –0.3469)	Beta	Yu et al,^28^ 2013
QALY difference^b^	QALY difference for intervention group vs control group in first 3 cycles	0.0420 (NA)	NA	Augustovski et al,^15^ 2018
Other				
High-risk proporition	Proportion of patients initiating at high risk	0.60 (0.45 to 0.75)	Beta	HCPIA trial^13,15^
Risk reduction^c^	Relative risk reduction of CVD events in intervention group	0.88 (0.99 to 0.79)	Log normal	Ettehad et al,^29^ 2016; Lewington et al,^8^ 2002
Discount^d^	Discount rate	0.05 (0.01 to 0.10)	NA	Augustovski, et al,^15^ 2018
Age^d^	Starting age, y	55 (40 to 70)	NA	HCPIA trial^13,15^
BP^b^	BP decrease per 18 mo, mm Hg	–5.30 (–10.34 to –0.27)	NA	Augustovski et al,^15^ 2018

Abbreviations: BP, blood pressure; CVD, cardiovascular diseases; INT $, international dollars; NA, not applicable; QALY, quality-adjusted life-year.

^a^ Ranges are either 95% CIs or values within 25% of base-case value.

^b^ QALY difference and BP decrease per 18 months were not included in the sensitivity analysis.

^c^ Relative risk reduction of CVD was calculated based on formula: relative risk = exp([ln{0.8}/10 mm Hg] × effect size).

^d^ Discount rate and starting age were not included in the probabilistic sensitivity analysis.

### Modeling Sources of Clinical Outcomes and QALYs

At baseline, the probability of having a stroke or a CVD event was calculated using the Framingham 10-year risk equations for stroke and CVD and converted to a 6-month risk estimate for each cycle in the 10-year horizon.^26,31^ Based on the 10-year CVD risk estimated from trial data and a cutoff of 20%, patients were assigned to low-risk and high-risk health states in the base case. Transition probabilities governing movement between health states were either imputed from trial data or obtained from published sources.^27^

The primary measures of health benefits for this study included QALYs. In the HCPIA trial, QALYs were estimated from the EuroQol 5-dimension questionnaire, 3-level version and their associated utility score at both baseline and 18 months.^32^ QALYs at 6 months in the model were estimated from the within-trial period. The change in the utility per patient in the 18-month period was assumed to be linear. The utilities at baseline and 18 months were used to estimate the linear association, and they were further used to estimate the utility at 6 months.^16^

The relative risk reduction of CVD events was estimated based on decreased SBP difference between the intervention group and control group. According to Ettehad et al,^29^ every 10 mm Hg SBP reduction is associated with a significant decrease in the risk of major CVD events (relative risk [RR], 0.80 [95% CI, 0.77-0.83]). A corresponding HR and 95% CI was then calculated based on the effect size observed in the trial period. Also, as indicated in the Ettehad et al,^29^ the proportional risk reductions in major CVD will not differ by baseline disease history. Thus, the same RR estimates were implemented in the low- and high-risk groups.^29^

The quality-of-life weight of a CVD event was estimated based on the literature.^28,29^ All transition probabilities, utility inputs, and other data input sources are shown in Table 2. Half-cycle correction was applied to model costs and outcomes.

### Model Assumptions

The model combines a decision tree with a Markov model to estimate the cost-effectiveness of the hypertension program. A number of underlying assumptions were adopted for the base-case model. First, individuals scoring greater than the 10-year CVD risk threshold (ie, 20%) were considered as having high risk at baseline. In the subsequent cycles, patients in the low-risk group who developed a CVD would move to the high-risk group. The starting prevalence of patients in the high-risk or low-risk groups was assumed to be independent of intervention or control cohorts, given that the parent trial showed that baseline BP was adequately balanced through randomization.^13^ Second, irrespective of baseline risk, patients who survived after a CVD event started a new cycle in the high-risk group. Third, we did not establish separate health states for patients who had a second CVD event different from the first. This approach is justifiable and would make this model conservative, ie, underestimating the effectiveness of the multicomponent intervention program. Fourth, the risk of having a CVD event was assumed to vary across the 2 groups. Three scenarios were modeled to extrapolate the long-term trajectory indicated in the HCPIA trial. In the intermediate scenario (ie, the base case), the difference of effectiveness data between groups remained constant within a 5-year window and disappeared at 5 years of follow-up. In the optimistic scenario, the effectiveness of the intervention was extrapolated beyond the 18 months of the trial to a lifetime horizon. That is, patients’ effectiveness data of lowering the SBP during 18-month trial period was maintained over the patients’ lifetime. In the pessimistic scenario, the effectiveness data would only be applied for the trial period of 18 months. Fifth, age-specific life tables were used to determine the mortality rate at different ages, but no gender distinction was made.^33^ The transition probabilities of surviving a CVD event (ie, mortality rate of CVD event) was assumed to be independent of the treatment cohort. Finally, a 5% discount rate was applied to both costs and utilities at base-case analysis.

### Statistical Analysis

QALYs were the main measures of efficacy outcomes. The ICER between the multicomponent intervention and usual care was calculated using the difference in costs divided by the difference in effectiveness (in QALYs).

Methodological and individual parameter uncertainty (from the model structure, selection of data inputs, or other assumptions) was addressed by 1-way sensitivity analysis. A tornado diagram analysis was used to assess the relative weight of each variable on overall uncertainty. Values for sensitivity analyses were chosen either as systematic variations around the values in the primary model or from differences between observed data from the trial and published studies. The parameters tested by 1-way sensitivity analysis included time horizon, discount rate for costs and QALYs, mortality rate, transition probabilities, relative risk of CVD events, CVD event costs, and intervention costs. The ranges for 1-way sensitivity analyses were based on 95% CIs, when available. For unavailable ranges, a plausible range for values (ie, ±25%) was used in the 1-way sensitivity analysis.

Probabilistic sensitivity analysis (PSA) was used to assess the level of parameter uncertainty (from uncertainty and/or variance in the data inputs). Each model parameter (event probability, cost, or quality-of-life weight) was assigned to a base value and a distribution of possible values. Second-order Monte Carlo simulation was used to estimate mean expected costs and outcomes, and statistical measures of expected variance around the mean for each of 5000 iterations drawn from the distributions defined. A theoretical willingness-to-pay (WTP) threshold was set at INT $18 000, corresponding to the gross domestic product (GDP) of Argentina in international dollars for 2017. A cost-effectiveness acceptability curve (CEAC) was plotted to show the proportion of bootstrapped simulations in which the net benefit of the intervention was greater than 0 for at WTP. All analyses were done with TreeAge Pro Healthcare 2020 (TreeAge Software, Inc) and SAS version 9.4 (SAS Institute Inc). Baseline characteristics (eTable in the Supplement) were compared using 2-sample *t *tests for continuous variables and χ^2^ tests for categorical variables, with a statistical significance level of *P* < .05.

## Results

### Base-Case Analyses

In the base case, a cohort similar to that of the clinical trial was analyzed. There were 743 participants in the intervention group (349 [47.0%] men), with a mean (SD) age of 56.2 (12.0) years, and 689 participants in the control group (311 [45.1%] men), with a mean (SD) age of 56.2 (11.7) years. The eTable in the Supplement shows the characteristics of patients in the HCPIA trial at baseline, 6-month follow-up, and 18-month follow-up. Other patient characteristics and trial results were detailed in our previous study results.^13^ The 1-time intervention cost of INT $53.88 within 6 months of follow-up was derived from previously reported 18-month costs.^15^ The intervention and nonintervention costs related to health service resource use within 6 months derived from the 18-month intervention program period are presented at Table 1. Costs of health service utilization not related to the intervention were INT $105.30 in the intervention group and INT $82.69 in the usual care group. The difference in this cost was attributable to additional hospitalizations, outpatient care, antihypertensive medication use, and longer hospitalization stay. Other parameters included in the models are summarized in Table 2.

Results on total costs, effectiveness, and incremental costs and effectiveness are shown in Table 3. Given that the trial had an 18-month follow-up period, we extrapolated long-term effects using 2 extreme assumptions and 1 intermediate assumption for the base-case analysis. In the intermediate (base-case) analysis, the multicomponent program was more beneficial than usual care (8.42 QALY vs 8.29 QALY) and also more expensive (INT $3096 vs INT $2473), resulting in an ICER of INT $4907/QALY gained.

**Table 3.** Results for the Base-Case and 2 Scenario Analyses Considering Different Long-term Benefit Extrapolation on Lifetime Horizon

Strategy	Cost, INT $	Incremental cost, INT $	Mean effect	Incremental effect	Cost/effect	ICER
Intermediate scenario, ie, base case						
Usual care	2472.61	NA	8.29 QALY	NA	298.30 INT $/QALY	NA
Multicomponent intervention	3095.62	623.01	8.42 QALY	0.13 QALY	367.82 INT $/QALY	4906.87 INT $/QALY
Optimistic scenario						
Usual care	2472.61	NA	8.29 QALY	NA	298.30 INT $/QALY	NA
Multicomponent intervention	3059.26	586.64	8.47 QALY	0.18 QALY	361.34 INT $/QALY	3306.08 INT $/QALY
Pessimistic scenario						
Usual care	2472.61	NA	8.29 QALY	NA	298.30 INT $/QALY	NA
Multicomponent intervention	3109.21	630.59	8.39 QALY	0.10 QALY	370.70 INT $/QALY	6473.90 INT $/QALY

Abbreviations: ICER, incremental cost-effectiveness ratio; INT $, international dollars; NA, not applicable; QALY, quality-adjusted life-year.

### Sensitivity Analyses

In the optimistic scenario, in which patients’ SBP improvement would be extrapolated outside the trial period following the mean decreasing rate during the trial period, the multicomponent hypertension intervention program yielded 8.47 discounted QALYs and accrued INT $3059 in discounted costs, while usual care yielded 8.29 discounted QALYs and accrued INT $2473 in discounted costs, with an ICER of INT $3306/QALY gained. In the pessimistic model, it was assumed that patients’ SBP improvement in the intervention group would only be applied in the 18-month trial period, and it would stay constant after the trial period ended. In this model, the multicomponent program was also more expensive compared with usual care (INT $3109 vs INT $2473), had higher QALYs (8.39 QALY vs 8.29 QALY), and had an ICER of INT $6474/QALY gained (Table 3).

**Figure 2.**
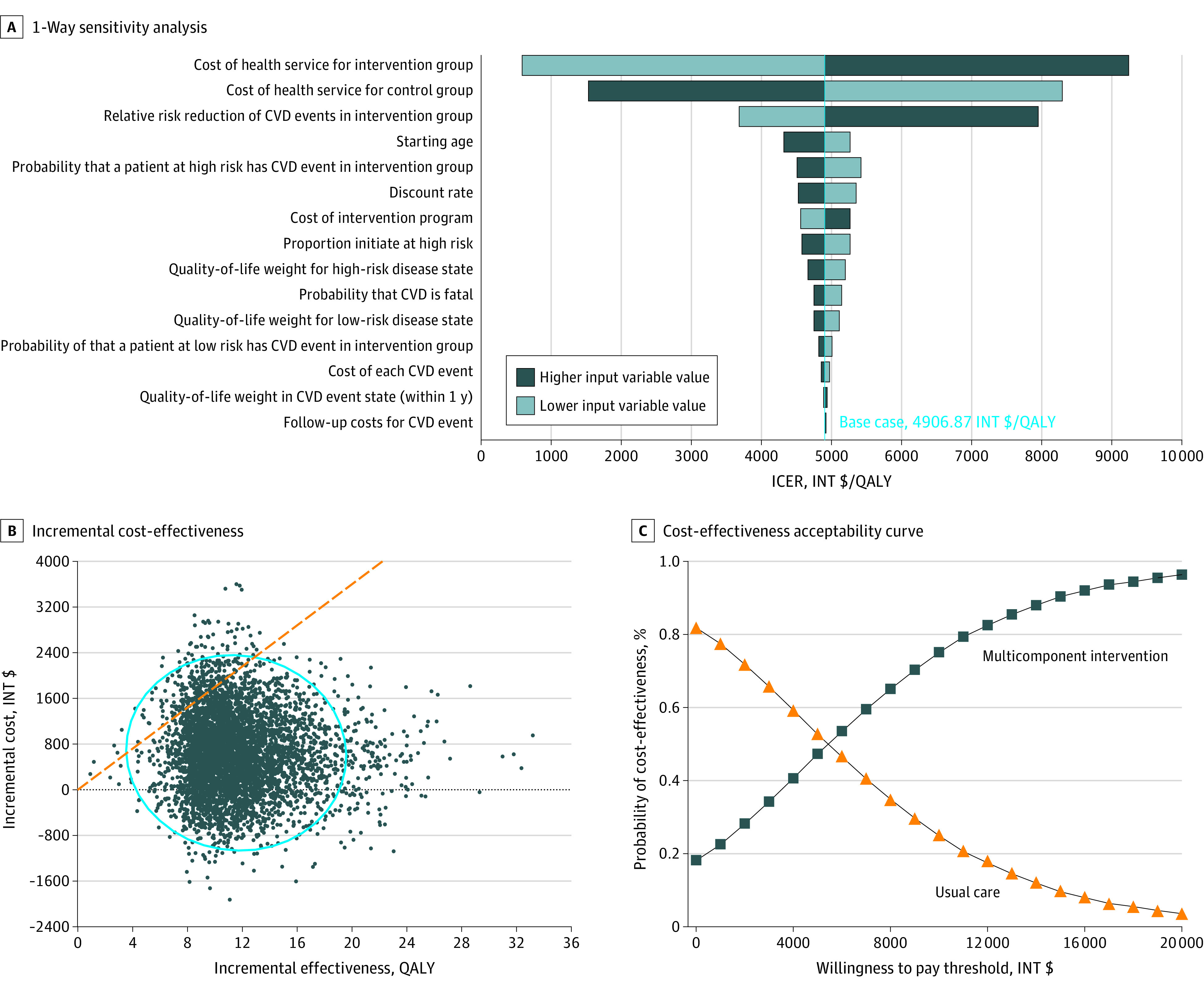
Sensitivity Analysis A, The model is most sensitive to cost of health service for the intervention and control groups and the relative risk of cardiovascular disease (CVD) between the 2 groups. B, Each dot represents the result of an iteration of 5000 total iterations. The blue circle indicates the 95% CI of results. The dashed diagonal line shows the willingness-to-pay threshold of international $18 000/quality-adjusted life-year.

One-way sensitivity analyses were conducted to assess the sensitivity of the model results to variations in model assumptions and inputs. The model results remained robust in sensitivity analyses, and the model was most sensitive to parameters of program costs and relative risk of CVD between the 2 groups in the base case (Figure 2A). Notably, all ICERs were less than the WTP threshold of INT $18 000/QALY during 1-way sensitivity analysis. Bootstrap methods were used to plot the uncertainty surrounding the estimates of expected incremental cost and expected incremental effect associated with the multicomponent intervention compared with usual care strategies for the management of hypertensive patients (Figure 2B). The CEAC in Figure 2C shows the probability of the multicomponent program being cost-effective compared with usual care in a range of WTP thresholds. Considering a discount rate of 5%, at a WTP of INT $18 000/QALY (corresponding to Argentina’s GDP for 2017), the multicomponent intervention had 99% probability of being cost-effective. Similarly, in 5000 probabilistic simulations, there was an approximately 94% probability that the multicomponent hypertension intervention program was cost-effective at a WTP threshold of INT $18 000/QALY. In general, the curves indicate the probability (based on the data in the model) that the intervention provides the greatest net benefit at different WTP thresholds to gain a QALY.

## Discussion

In this study, we developed a Markov model that integrated patient-level data from the HCPIA trial to assess the long-term cost-effectiveness of this hypertension management program in Argentina. Specifically, we constructed a lifetime Markov model to evaluate the cost-effectiveness of a multicomponent intervention for hypertension control by using patient-level clinical trial data and parameter inputs from the literature. To our knowledge, limited studies have reported trial-based long-term cost-effectiveness evaluation in the low-income setting in Argentina. Intensive hypertension control entailed more frequent office visits, laboratory tests, and greater medication use than did usual care and thus was more costly early on. However, these costs were balanced by health gains from prevented CVD events and deaths. This analysis suggests that this multicomponent hypertension management program was more effective than usual care at a very reasonable incremental cost. The results of the sensitivity analyses showed that cost-effective results were robust against wide assumptions, with ICERs remaining well below 1 GDP/QALY, a threshold widely used as a benchmark, in all analyses.^34^

A systematic review of cost-effectiveness evaluations of CHW interventions in the United States indicated that these interventions are cost-effective when used for targeted outreach for populations with increased risk.^35^ All interventions presented cost data of the CHW intervention through cost analysis, itemized costs of the intervention, and hourly wages. Another cost-effectiveness analysis of intensive BP management found that intensive BP management cost US $23 777/QALY gained.^36^ In that study, intensive BP management was defined as treatment of hypertension to an SBP goal of 120 mm Hg and standard BP management was defined as treatment of hypertension to an SBP goal of 140 mm Hg. In Argentina, CHWs are integrated into the primary care team, which facilitated the recruitment and training for this trial as well as the high adherence rate to the intervention. The findings of this study are consistent with the systematic review finding that CHW-led interventions are cost-effective among the target population. In addition, we included in the analysis varied costs, such as the costs for training activities for the CHWs on the participants’ intervention; training activities for physicians focused on standard treatment algorithms for stepped-care management based on clinical guidelines; BP monitors for all patients with hypertension for weekly measurement; and the compensation for CHWs on education, motivation, social support, and promoting health care utilization for participants and their families.

### Limitations

This study has limitations. External validity or generalizability of the findings is the first major concern. Our simulations represent a range of hypothetical treatment effects projected beyond the trial period, given that long-term data on treatment effects of intensive control vs standard control beyond the end of the trial are not available. The study was based on public clinic populations from LMICs. Results should not be extrapolated to other health care settings or high-income populations. Second, according to the ISPOR Task Force report,^37^ well-established, published models or those developed specifically for the trial are recommended for projecting costs and outcomes in trial-based economic evaluation. For this study, we developed the Markov model specifically for the trial and calibrated and internally validated the modeled outcomes with the observed within-trial outcomes. Due to a lack of long-term epidemiological data for patients in Argentina with hypertension, we did not perform other validation. Nevertheless, long-term cardiovascular risk uses the widely used Framingham risk equations that, although not uncontroversial, are common currency. Third, both the components of the cost and effectiveness estimates are likely to be affected by sources of systematic uncertainty. Due to different study designs and patient characteristics, these cost and utility inputs might not be directly comparable with the current study setting. However, we would expect the effect of these limitations to be minimal, and model robustness could be partially examined by the sensitivity analyses. Fourth, the definition of high risk vs low risk is crude, based on a cutoff of 20%. For the patients with a CVD risk score extremely close to this cutoff (eg, 21% vs 19%), those considered high risk would have 4 times higher risk compared with those considered low risk, even though their risk scores do not differ significantly. In addition, in our analysis, it was assumed that there was no gender distinction in the mortality rate and that CVD risk is independent of age. However, this may not be true. In addition, the assumed optimistic scenario that the benefit of CVD risk reduction extends through the lifetime horizon may not be sufficiently realistic in practice. However, with this assumption, the current analysis could provide an upper bound for the potential benefits of the multicomponent intervention regarding hypertension control. Additionally, as with all economic models, results from this cost-effectiveness analysis were contingent on the assumptions for the Markov model.

## Conclusions

The study results aligned with the results of the within-trial cost-effectiveness analysis and additionally found that the intervention was cost-effective when extrapolating results to a longer-term time horizon in different and heterogeneous scenarios. The multicomponent intervention adopted in the HCPIA trial was a cost-effective strategy to improve hypertension management and reduce the risk of associated CVD in Argentina. The findings of this study also support the idea that similar multicomponent intervention programs could potentially be an efficient use of health care resources in other LMICs.

## Notes

Supplement. **eTable.** Characteristics of Patients in Argentina Study
