# Supplementary material for: Cost-effectiveness of a Multicomponent Intervention for Hypertension Control in Low-Income Settings in Argentina
Source: JAMA Netw Open. 2021 Sep 14;4(9):e2122559. doi: 10.1001/jamanetworkopen.2021.22559 (PMC8441594; doi:10.1001/jamanetworkopen.2021.22559)
Supplement: Supplement. — eTable. Characteristics of Patients in Argentina Study [file jamanetwopen-e2122559-s001.pdf]

Supplemental Online Content

Zhang Y, Yin L, Mills K, et al. Cost-effectiveness of a multicomponent intervention for hypertension control in low-income settings in Argentina. *JAMA Netw Open.* 2021;4(9):e2122559. doi:10.1001/jamanetworkopen.2021.22559

eTable. Characteristics of Patients in Argentina Study

This supplemental material has been provided by the authors to give readers additional information about their work.

© 2021 Zhang Y et al. *JAMA Network Open.*

eTable

Characteristics[1]	Baseline						
	Intervention group			Control group			p-value
	N*			N*			
Male, n (%)	*743*	349	46.97%	*689*	311	45.14%	0.5205
Mean age in years (SD)	*743*	56.3	-11.95	*689*	56.17	-11.69	0.8365
Mean blood pressure in mmHg (SD)							
SBP	*743*	151.73	-16.81	*689*	149.86	-16.02	0.0321*
DBP	*743*	92.03	-11.5	*689*	89.92	-12.03	0.0007*
Well-controlled patients, n (%) [2]	*743*	159	21.40%	*689*	159	23.08%	0.4455
Patients with diabetes mellitus, n (%)[3]	*742*	175	23.58%	*689*	146	21.19%	0.2779
Mean body mass index, kg/m^2 (SD)	*742*	31.77	-6.15	*687*	31.44	-6.11	0.3096
Smoker, n (%)	*742*	144	19.41%	*689*	134	19.45%	0.9842
Patients under antihypertensive pharmacological treatment, n (%)	*743*	639	86.00%	*689*	575	83.45%	0.1798
Framingham CVD10-year risk score, (SD)[4]	*740*	0.29	-0.19	*687*	0.28	-0.19	0.1222
Cardiovascular risk (High risk >20%), n (%)	*740*	459	62.03%	*687*	405	58.95%	0.235
	**6 Months**						
	Intervention group			Control group			p-value
	N*			N*			
Male, n (%)	*743*	349	46.97%	*689*	311	45.14%	0.5205
Mean age in years (SD)	*743*	56.8	-11.95	*689*	56.67	-11.69	0.8365
Mean blood pressure in mmHg (SD)							
SBP	*722*	140.1	-18.84	*682*	142.4	-21.07	0.0274*
DBP	*722*	85.45	-13.16	*682*	86.38	-14.11	0.2026
Well-controlled patients, n (%) [2]	*743*	354	47.64%	*689*	284	41.22%	0.0145*
Patients with diabetes mellitus, n (%)[3]	*742*	175	23.58%	*689*	146	21.19%	0.2779
Mean body mass index, kg/m^2 (SD)	*720*	31.78	-6.28	*679*	31.4	-6.05	0.25
Smoker, n (%)	*722*	140	19.39%	*682*	133	19.50%	0.9582
Patients under antihypertensive pharmacological treatment, n (%)	*661*	627	94.86%	*623*	575	92.30%	0.0607
Framingham CVD10-year risk score, (SD)[4]	*658*	0.27	-0.19	*618*	0.26	-0.19	0.969

© 2021 Zhang Y et al. *JAMA Network Open.*

Cardiovascular risk (High risk >20%), n (%)	*658*	353	53.65%	*618*	325	52.59%	0.705
	**18 Months**						
	**Intervention group**				**Control group**		**p-value**
	**N***			**N***			
Male, n (%)	*743*	349	46.97%	*689*	311	45.14%	0.5205
Mean age in years (SD)	*743*	57.8	-11.95	*689*	57.67	-11.69	0.8365
Mean blood pressure in mmHg (SD)							
SBP	*709*	132.7	-15.72	*648*	137.2	-17.6	<.0001*
DBP	*709*	79.74	-10.62	*648*	83.3	-11.76	<.0001*
Well-controlled patients, n (%) [2]	*743*	551	74.16%	*689*	381	55.30%	<.0001*
Patients with diabetes mellitus, n (%)[3]	*708*	115	16.24%	*647*	118	18.24%	0.331
Mean body mass index, kg/m^2 (SD)	*708*	32.02	-6.52	*646*	31.76	-6.21	0.4445
Smoker, n (%)	*709*	124	17.49%	*648*	118	18.21%	0.7291
Patients under antihypertensive pharmacological treatment, n (%)	*660*	629	95.30%	*592*	542	91.55%	0.0071*
Framingham CVD10-year risk score, (SD)[4]	*658*	0.24	-0.18	*589*	0.25	-0.17	0.2522
Cardiovascular risk (High risk >20%), n (%)	*658*	311	47.26%	*589*	307	52.12%	0.0867

Notes:
[1] N* denotes the number of non-missing observations.
[2] Well controlled patients were defined as those SBP <140 mmHg and DBP <90 mmHg.
[3] In the 6-month visit, there's no data collected for diabetes status, the status was carried over from baseline.
[4] The 10-year risk score were estimated based on Framingham risk equation.
*Denotes p-value <0.05

© 2021 Zhang Y et al. *JAMA Network Open.*
